# Maternal and neonatal risks and outcomes after bariatric surgery: a comparative population based study across BMI categories in Qatar

**DOI:** 10.1038/s41598-024-69845-y

**Published:** 2024-11-07

**Authors:** Nader I. Al-Dewik, Muthanna Samara, Adel Mahmah, Aseel Al-Dewik, Seba Abou Nahia, Hana J. Abukhadijah, Yahya Samara, Sara Hammuda, Aleem Razzaq, Manar R. Al-Dweik, Asma Alahersh, Lina Moamed, Rajvir Singh, Sawsan Al-Obaidly, Tawa Olukade, Mohamed A. Ismail, Alaa Alnaama, Binny Thomas, John Paul Ben Silang, Gheyath Nasrallah, Nasser Rizk, MWalid Qoronfleh, Usama AlAlami, Thomas Farrell, Palli Valapila Abdulrof, Mai AlQubaisi, Hilal Al Rifai

**Affiliations:** 1 Department of Research, Women’s Wellness and Research Center, Hamad Medical Corporation, P.O.BOX. 3050, Doha, Qatar; 2 Genomics and Precision Medicine (GPM), College of Health & Life Science (CHLS), Hamad Bin Khalifa University (HBKU), 34110 Doha, Qatar; 3 Translational Research Institute (TRI), Hamad Medical Corporation (HMC), Doha, Qatar; 4 Faculty of Health and Social Care Sciences, Kingston University, St. George’s University of London, London, UK; 5 Department of Psychology, Kingston University London, Kingston upon Thames, UK; 6 Health Profession Awareness Program, Health Facilities Development, Hamad Medical Corporation (HMC), Doha, Qatar; 7 School of Medicine, Bahcesehir University, Istanbul, Turkey; 8 Department of Academic Health System, Hamad Medical Corporation (HMC), Doha, Qatar; 9 Faculty of Life Sciences & Medicine, King‘s College London, London, UK; 10 Hamad Medical Education, Hamad Medical Corporation (HMC), Doha, Qatar; 11 Cardiology Research Center, Heart Hospital, Hamad Medical Corporation (HMC), Doha, Qatar; 12 Obstetrics and Gynecology Department, Women’s Wellness and Research Center, Hamad Medical Corporation (HMC), Doha, Qatar; 13 Department of Pediatrics and Neonatology, Neonatal Intensive Care Unit, Newborn Screening Unit, Women’s Wellness and Research Center, Hamad Medical Corporation (HMC), Doha, Qatar; 14 Department of Pharmacy, Women’s Wellness and Research Center, Hamad Medical Corporation (HMC), Doha, Qatar; 15 Department of Biomedical Science, College of Health Sciences, Member of QU Health, Qatar University, Doha, Qatar; 16 Q3CG Research Institute (QRI), Research & Policy Division, 7227 Rachel Drive, Ypsilanti, MI 48917 USA; 17 21HealthStreet, Consulting Services, 1 Christian Fields, London, SW16 3JY UK; 18 School of Life Science, Manipal Academy of Higher Education (MAHE), Dubai, United Arab Emirates

**Keywords:** Body Mass Index (BMI), Obesity, Post-bariatric surgery, Pregnancy outcomes, Neonatal outcomes, Gestational diabetes, Health care, Paediatric research

## Abstract

The impact of Bariatric Surgery (BS) on maternal and neonatal outcomes among pregnant women is not fully understood, especially in comparison to different weight categories. The primary aim of this study is to assess the factors associated to women who have undergone BS. The study also investigates the maternal and neonatal outcomes amongst this group in comparison to the three Body Mass Index (BMI) groups (women with obesity, overweight and normal weight). A 12-month population-based retrospective study was conducted using registry data from the PEARL-Peristat Study at the Women's Wellness and Research Center (WWRC) in Qatar from January 1, 2017, through December 31, 2017. Both univariate and multivariable regression analyses were employed to scrutinize risk factors and maternal and neonatal outcomes. The study included 6212 parturient women, of which 315 had a history of BS, while 5897 with no BS history. Qatari women, aged 35 and higher, with parity > 1, diabetes, and hypertension were more likely to be in the post-BS group. Women in the post-BS group were found to be more likely to have a cesarean delivery (37.5% vs. 24%, Adjusted Odds Ratio (aOR) = 1.59, CI 1.18–2.14), preterm babies (10% vs. 7%, aOR = 1.66, CI 1.06–2.59), and stillbirth (1.6% vs. 0.4%, aOR = 4.53, CI 1.33–15.50) compared to the normal weight women group. Moreover, post-BS women had a higher risk of low-birth-weight neonates than women with obesity (15% vs. 8%, aOR = 1.77, CI 1.153–2.73), overweight (15% vs. 7%, aOR = 1.63, CI 1.09–2.43), and normal weight (15% vs. 8%, aOR = 1.838, CI 1.23–2.75). Finally, women in the post-BS group were more likely to have low-birth-weight neonates amongst term babies than women with obesity and overweight. Pregnancies with post-BS should be considered a high-risk group for certain medical outcomes and should be monitored closely. These findings may guide the future clinical decisions of antenatal and postnatal follow-up for post-BS women**.**

## Introduction

Obesity is a major public health concern. In 2022, there were 2.5 billion overweight adults aged 18 and older, of whom more than 890 million were living with obesity. This means that 43% of the adult population, including 43% of men and 44% of women, were overweight. This marks a significant rise from 1990, when only 25% of adults were overweight, World obesity Atlas projections indicated that obesity will increase from 38% of the world’s population in 2020 to over 50% by 2035^1,2^. In 2012, a survey of 2496 adults in Qatar revealed that 70.1% were overweight, including 71.8% of men and 68.3% of women. Of these, 41.4% were obese, with obesity affecting 39.5% of men and 43.2% of women^3^.

Obesity is associated with several comorbidities including hypertension, musculoskeletal disorders, cancer, and type 2 diabetes^4,5^. Women with obesity of childbearing age experience additional complications such as polycystic ovarian syndrome (PCOS) and infertility^6–8^. Pregnant women with obesity and overweight have a higher incidence of gestational diabetes, preeclampsia^9^, spontaneous miscarriage^10^, labor induction, anesthesia-related complications, primary cesarean section, and perioperative morbidity plus longer hospital stays^11–13^. This leads to a substantial increase in healthcare costs.

Moreover, neonates of pregnant women with obesity face a higher risk of pre- and post-term birth complications such as small and large for gestational age (SGA/LGA), congenital anomalies^14^, and perinatal mortality^15–17^. Offspring of women with obesity may also experience health complications later in life, such as hypertension, diabetes or cardiovascular diseases^18^.

Weight reduction approaches to lower the risk of obesity complications for both mothers and neonates is challenging^15,16^. Bariatric surgery (BS) is one of the preferred procedure in women with severe obesity, demonstrating good health outcomes^19,20^. BS procedures are continually on the rise. Globally over 800,000 BS procedures have been performed across 61 countries and 17 national registries between 2014 and 2019^21^.

Qatar has been actively combating obesity. Since 2011, HMC has performed over 10,000 BS surgeries; of those, 703 took place in 2022. Notably, in 2012 a significant milestone was achieved when the first bariatric robotic surgery was performed^22^.

There are limited studies on perinatal outcomes other than the size for gestational age and preterm birth^23–25^. The primary aim of this study is to assess the medical risk factors, and pregnancy and neonatal outcomes of pregnant women who have undergone bariatric surgery in comparison to pregnant women without a history of BS in Qatar.

## Methods

### Study design and data collection

This study utilised a 12-month, population-based retrospective study, conducted from January to December 2017, based on electronic medical record registry data from the PEARL-Peristat Study at WWRC, Hamad Medical Corporation (HMC) in Qatar^26–32^. This population-based registry was designed using routinely collected hospital data for parturient women and their offspring.

The study consisted of all singleton live births at 24^+0^ weeks’ gestation and above.

We excluded multiple-birth pregnancies due to their association with a higher occurrence of complications and variations in fetal growth. Out of 16,248 pregnancies, our study focused on 6212 singleton births with available data on post-bariatric surgery (BS) and body mass index (BMI). Among these, 315 were women had undergone BS. To form a comparison group, we included pregnancies in women without a history of BS, categorised as women with obesity (n = 1918), overweight (n = 1953), and normal weight controls (n = 2026). There were no exclusion criteria applied to any of the groups. This study complies with the World Medical Association Declaration of Helsinki regarding for ethical research conduct and was approved by the Hamad Medical Corporation Institutional Review Board, with a waiver informed consent.

### Maternal factors

The *main outcome variable* was Body mass index (BMI), which was examined and calculated as pre-pregnancy BMI or BMI during early pregnancy [i.e., at the first prenatal visit, depending on the availability of the relevant data: gestational age (< 13 weeks)] amongst all women. Early/pre-pregnancy BMI (kg/m^2^) was calculated using the ratio of the pre-pregnancy or early booking weight (kg) divided by the measured height (m^2^). The derived early/pre-pregnancy BMI was grouped into four groups: underweight (≤ 18.5 kg/m^2^), normal weight (18.5 to 24.9 kg/m^2^), overweight (25 to 29.9 kg/m^2^), and women with obesity (≥ 30 kg/m^2^) following the NHLBI/WHO guidelines^19,33^. The women with obesity were then classified as women with a history of bariatric and women without a history of bariatric.

Accordingly, pregnant women were classified as having a normal weight, overweight, and women with obesity (with and without a history of bariatric surgery); underweight women were excluded as their inclusion was not relevant to the study's focus. Pregnancy outcomes were extracted as documented in the pregnant women notes or coding summaries.

*Maternal demographic* factors included age of the mother at delivery (< 35 years old vs. ≥ 35 years of age), and nationality (Qataris vs. other Arabs (based on the UNESCO list of Arab countries), and other nationalities). In addition, *pregnancy factors* included pregnancy mode (spontaneous vs. Assisted Reproductive Technology (ART) and Ovulation Induction (OI)), chronic/essential hypertension (yes vs. no) and if yes, pregnancy induced hypertension (PIH) (considered separately in the analyses), postpartum haemorrhage (PPH) (blood loss was defined as loss of ≥ 500 ml of blood for vaginal births or ≥ 1000 ml for caesarean delivery) and preeclampsia (PET); delivery mode (vaginal vs. caesarean) and if caesarean whether it was emergency vs. elective; and diabetes based on their glycemic status (none, overt diabetes mellitus (DM), and gestational diabetes mellitus (GDM)). For GDM analysis, women diagnosed with diabetes before pregnancy were excluded. In addition, the study included parity (nulliparous vs. parity 1–4 and parity ≥ 5). Parity classification was chosen to capture the specific demographic and clinical characteristics of our study population, which exhibits a high range of parity (0–11). Existing literature indicates that maternal and neonatal health outcomes vary significantly with parity levels. Higher parity (≥ 5) may be associated with increased risks of obstetric complications, and preterm birth, compared to lower parity (1–4). Additionally, high parity is linked with greater healthcare resource utilisation and different demographic characteristics, including maternal age and socioeconomic status. By using this classification, we ensure that our findings are comparable with other studies in the Middle East and reflective of the clinical realities in Qatar, providing relevant insights into maternal and neonatal health risks associated with varying parity levels^34,35^.

### Neonatal factors

Neonatal factors included data about gestational age at time of delivery (Full term: =  > 37 vs. extremely to very preterm: 24–31 weeks and moderate to late preterm: 32–36 weeks). Gestational age (GA) was based on the mother’s last menstrual period (LMP), early ultrasound scan (USS) and Ballard scoring^19,33^. The GA was classified in accordance with established international definitions^20,36^. In addition, the study included birth weight, which was classified into Low Birth Weight (LBW) (≤ 2499 g) and normal birth weight (≥ 2500 g) for the whole sample. Term births, those who were born at 37 weeks of gestational age or more, were also classified into LBW and normal birth weight, and included as a separate factor. Low Apgar scores at 1 and 5 min were defined as any score lower than 7. Finally, baby disposition, which was categorised into admission to a neonatal intensive care unit (NICU) and stillbirth (fetal death at ≥ 22 completed weeks of gestation) was also included.

### Statistical analysis

Statistical analysis was performed using IBM SPSS Statistics version 28 (IBM Corp., Armonk, NY, USA). Categorical and binary variables were reported as frequencies and percentages, while interval variables were presented as means and standard deviations. Chi-square tests were employed to examine the associations between maternal and neonatal factors in one hand, and BMI categories versus post-bariatric surgery (post-BS) status on the other hand (post-BS women versus normal weight, overweight, and women with obesity). The same analytical approach was applied to compare BMI categories (normal weight, overweight, and women with obesity).

**Risk Factors of post-BS:** Univariate logistic regression analyses were conducted to assess the associations between maternal and neonatal factors (Independent Variables “IVs”) and post-BS status versus each BMI category as an outcome (Dependent Variable “DV”). Additionally, univariate logistic regressions were performed for the comparisons between BMI categories as outcomes. Crude odds ratios (cOR) with 95% confidence intervals (CIs) were calculated and reported.

Multiple logistic regression analyses were subsequently conducted, including variables that were significant at p < 0.05 in the univariate analyses as IVs, and post-BS status versus each BMI category as an outcome (DV). The same analytical approach was applied when the BMI comparisons as the outcomes. The final models reported adjusted odds ratios (aOR) with 95% CIs for the independent risk factors. Statistical significance was defined as p < 0.05 (two-tailed).

**Outcomes of post-BS:** On the other hand, the same approaches were employed to investigate the outcomes of BS when compared to BMI groups. Univariate logistic regression analyses were conducted to evaluate the associations between post-BS status and each BMI category (IVs) and various pregnancy outcomes (DVs). Additionally, univariate logistic regressions were performed to compare different BMI categories as risk factor of pregnancy outcomes. Crude odds ratios (cOR) with 95% CIs were calculated and reported. Subsequently, multiple logistic regression analyses were conducted, incorporating variables that were significant at p < 0.05 in the univariate analyses from the first stage (Risk factors of post-BS stage) as IVs, and the pregnancy outcomes as DVs. The final models reported adjusted odds ratios (aOR) with 95% CIs for the independent risk factors. Statistical significance was defined as p < 0.05 (two tailed).

### Ethics approval

The study was approved by the Hamad Medical Corporation Institutional Review Board.

### Informed consent

The informed consent was waived by the Hamad Medical Corporation Institutional Review Board committee.

## Results

### Comparison analysis between the available data versus the missing data

A comparative analysis was conducted between cases with available data (n = 6212) and those with missing data on post-bariatric surgery (post-BS) and body mass index (BMI) (n = 10,036) concerning demographic and clinical variables. Significant differences were observed between these two groups. Data availability was higher among older mothers (≥ 35 years) compared to younger mothers (22.5% vs. 18.1%; p < 0.001). Additionally, essential hypertension was more prevalent amongst women who were included in the study (1.8% vs. 0.9%; p < 0.001). The mode of delivery also differed significantly, with cesarean delivery being more common among those with available data (32.5% vs. 28.4%; p < 0.001). Induced labor was more frequently reported in the data-available group (17.8% vs. 14.3%; p < 0.001), as was gestational diabetes mellitus (30.6% vs. 22.9%; p < 0.001). Elective Caesarean Section (CS) rates were higher among those with available data (52.5% vs. 48.3%; p < 0.01). Finally, the prevalence of Diabetes Mellitus (DM) was significantly greater in the data-available group (5.5% vs. 1.8%; p < 0.001). There were no significant differences in relation to the rest of the demographic and clinical factors.

### Characteristics of the study population

A total of 6212 women who had singleton pregnancies from January 2017 to December 2017 comprising of post-BS (n = 315/6212; 5.07%) were included in the study. These were compared to women with obesity (n = 1918/6212; 30.87%), overweight (n = 1953/6212; 31.44%) and normal weight pregnant women (n = 2026/6212; 32.61%) with no history of weight loss surgeries.

The clinical characteristics and outcomes of the studied population are listed in Table 1. It shows general differences between post-BS, women with obesity, overweight, and normal groups of pregnant women in relation to demographic and clinical factors.

**Table 1:** The clinical characteristics and outcomes of the overall study population.

	Total (n = 6212)		Post-bariatric		Obese		Overweight		Normal		P-value
					No surgery (n = 1918)		No surgery		No surgery		
			(n = 315)		(n = 1918)		(n = 1953)		(n = 2026)		
Maternal age (Mean ± SD)^a^	30.08 ± 5.6		31.7 ± 5.4		31.8 ± 5.6		30.3 ± 5.5		28.05 ± 5.1		** 0.000**
Maternal age											** 0.000**
< 35 years	4813	77.50%	217	68.90%	1309	68.20%	1485	76.0%	1802	88.90%	
≥ 35 years	1399	22.50%	98	31.10%	609	31.80%	468	24.0%	224	11.10%	
Conception mode											0.750
Spontaneous	5928/6155	96.30%	297/313	94.90%	1824/1902	95.90%	1855/1933	96.0%	1952/2007	97.26%	
OI	38/6155	0.60%	4/313	1.30%	10/1902	0.50%	16/1933	0.8%	8/2007	0.40%	
ART	189/6155	3.10%	12/313	3.80%	68/1902	3.60%	62/1933	3.2%	47/2007	2.30%	
Nationality											** 0.000**
Qatari	1860	29.90%	250	79.40%	626	32.60%	531	27.20%	453	22.40%	
Other Arabs	2592	41.70%	47	14.90%	893	46.60%	845	43.30%	807	39.80%	
Other Nationalities	1760	28.30%	18	5.70%	399	20.80%	577	29.50%	766	37.80%	
Parity											** 0.000**
Nulliparous	1676	27.00%	68	21.60%	321	16.70%	482	24.70%	805	39.70%	
Parity 1–4	4081	65.70%	204	64.80%	1360	70.90%	1340	68.60%	1177	58.10%	
Parity ≥ 5	455	7.30%	43	13.70%	237	12.40%	131	6.70%	44	2.20%	
Chronic/Essential HTN											** < 0.001**
HTN	110	1.80%	10	3.20%	67	3.50%	23	1.20%	10	0.50%	
No HTN	6102	98.20%	305	96.80%	1851	96.50%	1930	98.80%	2016	99.50%	
PET											**0.007**
Yes	140	2.30%	8	2.50%	61	3.20%	38	1.90%	33	1.6%	
No	6072	97.70%	307	97.50%	1857	96.80%	1915	98.10%	1993	98.40%	
Assisted birth											** 0.000**
Yes	374/4194	8.90%	8/197	4.10%	77/1126	6.80%	119/1329	9.00%	1701/542	11.0%	
No	3820/4194	91.1	189/197	95.9	1049/1126	93.2	1210	91	1372/1542	88.97%	
Delivery											** 0.000**
Vaginal	4194	67.50%	197	62.50%	1126	58.70%	1329/1329	68.00%	1542	76.10%	
Caesarean	2018	32.50%	118	37.50%	792	41.30%	624	32.00%	484	23.90%	
Induction of labor											**0.000**
Yes	747/4194	17.80%	32/197	16.20%	267/1126	23.70%	225/1329	16.90%	223/1542	14.50%	
No	3447/4194	82.20%	165/197	83.80%	859/1126	76.30%	1104/1329	83.10%	1319/1542	85.50%	
GDM											** 0.000**
Yes	1898	30.60%	60	19.00%	759	39.60%	631	32.30%	448	22.10%	
No	4314	69.40%	255	81.00%	1159	60.40%	1322	67.70%	1578	77.90%	
Diabetes											** 0.000**
No DM	4078	65.60%	244	77.50%	1030	53.70%	1255	64.30%	1549	76.50%	
GDM	1898	30.60%	60	19.00%	759	39.60%	631	32.30%	448	22.10%	
Overt DM	236	3.80%	11	3.50%	129	6.70%	67	3.40%	29	1.40%	
PIH											0.091
Yes	129	2.10%	6	1.90%	53	2.80%	34	1.74%	36	1.80%	
No	6083	97.90%	309	98.10%	1865	97.20%	1919	98.25%	1990	98.20%	
PPH*											0.317
Yes	323/6212	5.20%	18/315	5.70%	86/1918	4.50%	102/1953	5.20%	117/2026	5.80%	
No	5889/6212	94.80%	297/315	94.30%	1832/1918	95.50%	1851/1953	94.80%	1909/2026	94.20%	
Stillbirth											0.172
Yes	43	0.70%	5	1.60%	15	0.80%	14	0.70%	9	0.40%	
No	6169	99.30%	310	98.40%	1903	99.20%	1939	99.30%	2017	99.60%	
CS type											** 0.000**
Elective CS	1059/2018	52.50%	60/118	50.85%	451/792	56.94%	337/624	54.00%	221/494	44.74%	
Emergency CS	959/2018	47.50%	58/118	49.15%	341/792	43.05%	287/624	45.99%	273/494	55.26%	
Gestational age											**0.002**
24–31 weeks	76/6169	1.23%	6/310	1.90%	33/373	1.70%	16/1939	0.80%	21/2017	1.04%	
32–36 weeks	461/6169	7.50%	24/310	7.70%	168/373	8.80%	150/1939	7.70%	119/2017	5.90%	
Above 37 weeks	5632/6169	91.30%	280/310	90.30%	172/373	89.40%	1773/1939	91.40%	1877/2017	93.10%	
NICU											**0.005**
Yes	702/6161	11.40%	36/310	11.60%	255/1901	13.40%	213/1938	11.00%	198/2012	9.80%	
No	5459/6161	88.60%	274/310	88.40%	1646/1901	86,6%	1725/1938	89.00%	1814/2012	90.20%	
Preterm											**0.001**
Yes	537/6169	8.70%	30/310	9.70%	201/1903	10.60%	166/1939	8.60%	140/2017	6.90%	
No	5632/6169	91.30%	280/310	90.30%	1702/1903	89.40%	1773/1939	91.40%	1877/3017	93.10%	
Birth weight < 2500 g											**0.000**
Yes	506/6206	8.20%	47	14.90%	153/1915	8.00%	141/1951	7.20%	165/2025	8.10%	
No	5700/6206	91.80%	268	85.10%	1762/1915	92%	1810/1951	92.80%	1860/2025	91.90%	
Term low birth weight											** 0.000**
Yes	204/5644	3.60%	22/281	7.80%	46/1705	2.70%	51/1777	2.90%	85/1881	4.50%	
No	5440/5644	96.40%	259/281	91.20%	1659/1705	97.30%	1726/1777	97.10%	1796/1881	95.50%	
Low Apgar at 1 min											0.851
Yes	156/6143	2.50%	8/310	2.60%	50/1895	2.60%	44/1931	2.30%	54/2007	2.70%	
No	5987/6134	97.50%	302/310	97.40%	1845/1895	97.40%	1887/1931	97.70%	1953/2007	97.30%	
Low Apgar score at 5 min											**0.004**
Yes	17/6150	0.30%	4/310	1.30%	6/1898	0.30%	4/1930	0.20%	3/2008	0.15%	
No	6133/6150	99.70%	306/310	98.70%	1892/1898	99.70%	1930/1934	99.80%	2005/2008	99.85%	

Abbreviations: SD, Standard Deviation; OI, Ovulation Induction; ART, Assisted Reproductive Technology; PET, Pre-eclampsia; GDM, Gestational Diabetes Mellitus; DM, Diabetes Mellitus; PIH, Pregnancy-Induced Hypertension; PPH, Postpartum Hemorrhage; CS, Caesarean Section; NICU, Neonatal Intensive Care Unit. ^a^ Women with normal weight are significantly younger than the other three groups (women with post-BS, obesity and overweight) (p=0.000). Women with overweight are also significatly younger than women with post-BS and obesity (p=0.000).

Pregnant women aged ≥ 35 years and Qataris are more likely to be in the post-BS group. They were also more likely to give birth to babies with low birth weight (< 2500 g) and have low Apgar score at 5 min. Women with obesity had a higher prevalence of gestational diabetes mellitus (GDM), chronic/essential hypertension, induction of labor, NICU admission and cesarean delivery compared to the other groups. Pregnant women with obesity are also more likely to be aged ≥ 35 years compared to the non-surgery BMI groups. Also, preterm birth was found to be more likely amongst women with obesity, overweight, and post-BS groups compared to normal.

### Neonatal and maternal adverse events as risk factors for women in the post-bariatric surgery group compared to obesity, overweight, and normal weight groups

Univariate and multivariate regression analyses were performed to assess the risk factors for post-BS, women with obesity and overweight in comparison to normal weight (Table 2). The results show that Qatari women, aged 35 years and above, DM, parity (higher than 1), and Essential Hypertension (EHTN) were significantly more likely to be from the obesity group in comparison to the normal weight group even after adjustment for other significant factors from the univariate analysis. The same results were found for the overweight pregnant women (except for EHTN) and post-BS (except for DM) (see Table 2).

**Table 2:** Univariate (cOR) and multivariate regression (aOR) analyses of the risk factors associated with pregnant women weight status (post Bariatric surgery, obesity and overweight) vs. normal weight.

Risk factors	Obese				Overweight				Post-Bariatric			
	(*n* = 1159)				(*n* = 1953)				(*n* = 315)			
	cOR (95%CI)	p value	aOR^a^ (95%CI)	p value	cOR (95%CI)	p value	aOR^a^ (95%CI)	p value	cOR (95%CI)	p value	aOR^a^ (95%CI)	p value
Nationality												
Non Qatari	Ref				Ref				Ref			
Qataris	1.682 (1.460–1.938)	**0.000**	1.510 (1.258–1.813)	** 0.000**	1.297 (1.122–1.498)	** 0.000**	1.256 (1.058–1.491)	**0.009**	13.355 (9.971–17.888)	** 0.000**	15.567 (10.898–22.236)	** 0.000**
Maternal age												
< 35 years	Ref				Ref				Ref			
≥ 35 years	3.743 (3.161–4.431)	** 0.000**	2.080 (1.665–2.599)	** 0.000**	2.535 (2.132–3.015)	** 0.000**	1.831 (1.470–2.282)	** 0.000**	3.633 (2.757–4.788)	** 0.000**	2.154 (1.415–3.280)	** 0.000**
DM status												
No DM	Ref				Ref				Ref			
DM	6.690 (4.438–10.084)	** 0.000**	4.434 (2.863–6.866)	** 0.000**	2.852 (1.833–4.436)	** 0.000**	2.139 (1.344–3.403)	**0.001**	2.408 (1.187–4.883)	**0.015**	1.816 (0.763–4.321)	0.177
Parity												
Nulliparous	Ref				Ref				Ref			
Parity 1–4	2.898 (2.491–3.370)	** 0.000**	2.460 (2.034–2.975)	** 0.000**	1.901 (1.657–2.181)	** 0.000**	1.700 (1.442–2.004)	** 0.000**	2.052 (1.537–2.739)	** 0.000**	1.894 (1.327–2.703)	** 0.000**
Parity ≥ 5	13.508 (9.550–19.107)	** 0.000**	6.609 (4.228–10.330)	** 0.000**	4.972 (3.470–7.125)	** 0.000**	2.829 (1.788–4.477)	** 0.000**	11.569 (7.104–18.842)	** 0.000**	5.210 (2.556–10.620)	** 0.000**
Chronic/Essential HTN												
No HTN	Ref				Ref				Ref			
HTN	7.297 (3.744–14.224)	** 0.000**	4.211 (1.958–9.056)	** 0.000**	2.402 (1.141–5.061)	**0.021**	1.281 (0.539–3.045)	0.574	6.610 (2.729–16.012)	** 0.000**	4.578 (1.395–15.028)	**0.012**

Abbreviations: cOR, crude Odds Ratio; aOR, adjusted Odds Ratio; CI, Confidence Interval; Ref, reference group; DM, Diabetes Mellitus; HTN, Hypertension.

**Bold** values denote statistical significance.

^a^Adjusted for the significant variables from the univariate analysis (cOR).

When comparing post-BS with overweight pregnant women, it was found that that post-BS were significantly more likely to be Qataris and to have EHTN before and after adjustment. On the other hand, pregnant women with obesity in comparison to overweight were more likely to have multiple births (1–4 and > 5), DM, and EHTN even after adjustment for other significant factors from the univariate analysis. Finally, post-BS pregnant women were significantly more likely to be Qataris and less likely to be DM in comparison to pregnant women with obesity even after adjustment for other significant factors from the univariate analysis (see Table 3).

**Table 3:** Univariate (cOR) and multivariate (aOR) regression analyses of the risk factors associated with pregnant women weight status (post bariatric surgery, obesity and overweight).

Risk factors	Post-Bariatric vs. overweight				Obesity vs. overweight				Post-Bariatric vs. obese			
	(n = 315)				(*n* = 1918)				(*n* = 315)			
	cOR (95%CI)	P value	aOR^a^ (95%CI)	P value	cOR (95%CI)	P value	aOR^a^ (95%CI)	P value	cOR (95%CI)	p value	aOR^a^ (95%CI)	p value
Nationality												
Non-Qatari	Ref				Ref		Ref		Ref			
Qataris	10.300 (7.703–13.772)	** 0.000**	10.125 (7.547–13.582)	** 0.000**	1.298 (1.130–1.490)	** 0.000**	1.161 (0.975–1.384)	0.094	7.938 (5.945–10.599)	**0.000**	10.109 (7.118–14.357)	** 0.000**
Maternal age												
< 35 years	Ref	Ref			Ref		Ref		Ref			
≥ 35 years	1.433 (1.105–1.859)	**0.007**	1.148 (0.841–1.566)	0.834	1.476 (1.281–1.701)	** 0.000**	1.093 (0.898–1.331)	0.374	0.971 (0.751–1.255)	0.821	NA	
Parity												
Nulliparous	Ref				Ref		Ref		Ref			
Parity 1–4	1.079 (0.805–1.447)	0.611	1.019 (0.740–1.404)	0.908	1.524 (1.299–1.789)	** 0.000**	1.457 (1.192–1.781)	** 0.000**	0.708 (0.524–0.956)	**0.024**	0.787 (0.541–1.143)	0.208
Parity ≥ 5	2.327 (1.517–3.569)	** 0.000**	1.166 (0.703–1.934)	0.553	2.717 (2.103–3.509)	** 0.000**	2.085 (1.461–2.975)	** 0.000**	0.856 (0.564–1.300)	0.467	0.856 (0.504–1.453)	0.565
Diabetes status												
No DM	Ref	Ref			Ref		Ref		Ref			
DM	0.844 (0.440–1.621)	0.611			2.346 (1.727–3.187)	** 0.000**	1.880 (1.365–2.590)	** 0.000**	0.360 (0.191–0.677)	**0.002**	0.262 (0.136–0.505)	** 0.000**
Chronic/Essential HTN												
No HTN	Ref		Ref		Ref		Ref		Ref			
HTN	2.751 (1.297–5.837)	**0.008**	2.874 (1.202–6.870)	**0.031**	3.037 (1.883–4.898)	** 0.000**	2.832 (1.576–5.090)	** 0.000**	0.906 (0.461–1.780)	0.774	NA	

Abbreviations: cOR, crude Odds Ratio; aOR, adjusted Odds Ratio; CI, Confidence Interval; Ref, reference group; DM, Diabetes Mellitus; HTN, Hypertension. Significant values are in **bold**. ^a^ Adjusted for the significant variables from the univariate analysis.

### Outcomes for pregnant women post-BS, women with obesity, and overweight

Univariate and multivariate regression analyses were performed to investigate outcomes in pregnant women with post-BS history and based on weight status (women with obesity, overweight, and normal weight) (Table 4).

**Table 4:** Univariate (cOR) and multivariate (aOR) regression analyses of the outcomes for pregnant women with history of post-bariatric surgery and different weight statuses (obesity, overweight, and normal weight).

Outcomes	PET				Caesarean Section				PIH			
					(*n* = 2018)				(*n* = 129)			
	cOR (95%CI)	p value	aOR (95%CI)	p value	cOR (95%CI)	p value	aOR (95%CI)	p value	cOR (95%CI)	p value	aOR (95%CI)	p value
Post-BS vs normal^a^	1.574 (0.720–3.439)	0.256			1.908 (1.486–2.450)	** 0.000**	1.591 (1.182–2.143)	**0.002**	1.073 (0.449–2.568)	0.874		
Obese vs normal^a^	1.984 (1.293–3.044)	**0.002**	2.233 (1.254–3.976)	**0.006**	2.241 (1.955–2.569)	** 0.000**	1.772 (1.490–2.108)	**0.000**	1.571 (1.024–2.410)	**0.039**	1.562 (0.897–2.720)	0.115
Overweight vs normal^a^	1.198 (0.749–1.919)	0.451			1.496 (1.301–1.720)	** 0.000**	1.249 (1.055–1.478)	**0.010**	0.979 (0.610–1.572)	0.931		
Post-BS vs overweight^b^	1.313 (0.607–2.842)	0.489			1.276 (0.996–1.633)	0.054			1.096 (0.456–2.632)	0.838		
Obese vs overweight^c^	1.655 (1.099–2.495)	**0.016**	1.644 (0.943–2.865)	0.079	1.498 (1.313–1.709)	** 0.000**	1.407 (1.186–1.670)	**0.000**	1.604 (1.038–2.479)	**0.033**	1.381 (0.790–2.412)	0.257
Post-BS vs obese^d^	0.793 (0.376–1.674)	0.543			0.852 (0.666–1.089)	0.200			0.683 (0.291–1.603)	0.381		

Outcomes	PPH				Induction of labor (induced)				Assisted birth			
	(*n* = 323)				(*n* = 747)				(*n* = 374)			
	cOR (95%CI)	p value	aOR (95%CI)	p value	cOR (95%CI)	p value	aOR (95%CI)	p value	cOR (95%CI)	p value	aOR (95%CI)	p value
Post-BS vs normal^a^	0.989 (0.593–1.649)	0.966			1.147 (0.766–1.719)	0.506			0.342 (0.165–0.705)	**0.004**	0.500 (0.220–1.133)	0.097
Obese vs normal^a^	0.766 (0.576–1.019)	0.067			1.838 (1.509–2.240)	** 0.000**	1.757 (1.326–2.327)	**0.000**	0.592 (0.447–0.785)	** < 0.001**	0.778 (0.531–1.139)	0.196
Overweight vs normal^a^	0.899 (0.684–1.181)	0.445			1.205 (0.985–1.475)	0.069			0.794 (0.620–1.016)	0.066		
Post-BS vs overweight^b^	1.100 (0.657–1.842)	0.718			0.952 (0.635–1.426)	0.81			0.430 (0.207–0.895)	**0.024**	0.455 (0.208–0.994)	**0.048**
Obese vs overweight^c^	0.852 (0.635–1.143)	0.285			1.525 (1.251–1.860)	** 0.000**	1.522 (1.143–2.025)	**0.004**	0.746 (0.554–1.006)	0.055		
Post-BS vs obese^d^	1.291 (0.766–2.177)	0.338			0.624 (0.417–0.933)	**0.022**	0.607 (0.341–1.080)	0.089	0.577 (0.274–1.214)	0.147		

Outcomes	GDM				Stillborn				Low APGAR score at 1 min			
	(*n* = 1898)				(*n* = 43)				(*n* = 156)			
	cOR (95%CI)	p value	aOR (95%CI)	p value	cOR (95%CI)	p value	aOR (95%CI)	p value	cOR (95%CI)	p value	aOR (95%CI)	p value
Post-BS vs normal^a^	0.829 (0.614–1.119)	0.220			3.615 (1.204–10.856)	**0.022**	4.537 (1.329–15.494)	**0.016**	0.958 (0.451–2.033)	0.911		
Obese vs normal^a^	2.307 (2.007–2.651)	** 0.000**	2.177 (1.883–2.518)	**0.000**	1.767 (0.771–4.046)	0.178			0.980 (0.664–1.447)	0.920		
Overweight vs normal^a^	1.681 (1.460–1.937)	** 0.000**	1.611 (1.395–1.860)	**0.000**	1.618 (0.699–3.747)	0.261			0.843 (0.564–1.262)	0.407		
Post-BS vs overweight^b^	0.493 (0.366–0.663)	** 0.000**	0.573 (0.417–0.787)	**0.001**	2.234 (0.799–6.246)	0.125			1.136 (0.530–2.437)	0.743		
Obese vs overweight^c^	1.372 (1.203–1.565)	** 0.000**	1.344 (1.175–1.537)	**0.000**	1.092 (0.526–2.268)	0.814			1.162 (0.771–1.752)	0.473		
Post-BS vs obese^d^	0.359 ( 0.267–0.483)	** 0.000**	0.396 (0.291–0.540)	**0.000**	2.046 (0.738–5.670)	0.169			0.977 (0.459–2.082)	0.953		

Outcomes	Low Apagar score at 5 min				NICU admission				Birth weight < 2500 g			
	(*n* = 17)				(*n* = 702)				(*n* = 506)			
	cOR (95%CI)	p value	aOR (95%CI)	p value	cOR (95%CI)	p value	aOR (95%CI)	p value	cOR (95%CI)	p value	aOR (95%CI)	p value
Post-BS vs normal^a^	8.736 (1.946–39.223)	**0.005**	3.739 (0.517–27.059)	0.192	1.204 (0.826–1.755)	0.335			1.977 (1.395–2.801)	** < 0.001**	1.838 (1.230–2.747)	**0.003**
Obese vs normal^a^	2.119 (0.529–8.487)	0.289			1.419 (1.165–1.729)	**0.001**	1.238 (0.955–1.605)	0.107	0.979 (0.778–1.231)	0.855		
Overweight vs normal^a^	1.385 (0.310–6.197)	0.670			1.131 (0.922–1.388)	0.237			0.878 (0.695–1.110)	0.276		
Post-BS vs overweight^b^	6.307 (1.569–25.351)	**0.009**	3.468 (0.763–15.774)	0.108	1.064 (0.731–1.549)	0.746			2.251 (1.580–3.208)	** < 0.001**	1.631 (1.095–2.428)	**0.016**
Obese vs overweight^c^	1.530 (0.431–5.431)	0.510			1.255 (1.033–1.523)	**0.022**	1.090 (0.844–1.408)	0.509	1.115 (0.879–1.414)	0.371		
Post-BS vs obese^d^	4.122 (1.157–14.691)	**0.029**	2.069 (0.296–14.444)	0.463	0.848 (0.585–1.230)	0.385			3.063 (1.813–5.177)	** < 0.001**	1.773 (1.153–2.728)	**0.009**

Outcomes	Conception method: ART				Conception method: Ovulation induction				Preterm			
	(n = 59)				(n = 12)				(n = 566)			
	cOR (95%CI)	p value	aOR (95%CI)	p value	cOR (95%CI)	p value	aOR (95%CI)	p value	cOR (95%CI)	p value	aOR (95%CI)	p value
Post-BS vs normal^a^	0.596 (0.312–1.136)	0.116			0.304 (0.091–1.017)	0.053			1.570 (1.059–2.327)	**0.025**	1.662 (1.066–2.590)	**0.025**
Obese vs normal^a^	0.646 (0.443–0.942)	**0.023**	0.676 (0.406–1.126)	0.133	0.748 (0.294–1.898)	0.541			1.604 (1.285–2.001)	** 0.000**	1.320 (0.984–1.770)	0.064
Overweight vs normal^a^	0.720 (0.491–1.058)	0.094			0.475 (0.203–1.113)	0.087			1.285 (1.022–1.616)	**0.032**	1.282 (0.969–1.696)	0.083
Post-BS vs overweight^b^	0.827 (0.441–1.553)	0.555			0.640 (0.213–1.929)	0.428			1.222 (0.829–1.801)	0.312		
Obese vs overweight^c^	0.897 (0.632–1.272)	0.541			1.573 (0.712–3.476)	0.263			1.248 (1.011–1.541)	**0.039**	1.046 (0.791–1.383)	0.754
Post-BS vs obese^d^	0.923 (0.493–1.752)	0.801			0.407 (0.127–1.306)	0.131			0.979 (0.667–1.436)	0.913		

Outcomes	Gestational age (24–31 weeks)				Gestational age (32–36 weeks)				Term low birth weight (LBW)			
	(n = 46)				(n = 275)				(N = 204)			
	cOR (95%CI)	p value	aOR (95%CI)	p value	cOR (95%CI)	p value	aOR (95%CI)	p value	cOR (95%CI)	p value	aOR (95%CI)	p value
Post-BS vs normal^a^	0.359 (0.170–0.758)	**0.007**	0.430 (0.166–1.113)	0.082	0.753 (0.478–1.188)	0.223			1.795 (1.103–2.920)	**0.018**	1.435 (0.823–2.500)	0.203
Obese vs normal^a^	0.558 (0.334–0.933)	**0.026**	0.720 (0.371–1.394)	0.330	0.638 (0.501–0.813)	** 0.000**	0.802 (0.574–1.121)	0.196	0.586 (0.407–0.844)	**0.004**	0.528 (0.324–0.860)	**0.010**
Overweight vs normal^a^	1.031 (0.576–1.845)	0.919			0.742 (0.580–0.950)	**0.018**	0.755 (0.554–1.029)	0.075	0.624 (0.438–0.889)	**0.009**	0.755 (0.502–1.138)	0.18
Post-BS vs overweight^b^	0.348 (0.163–0.742)	**0.006**	0.290 (0.110–0.764)	**0.012**	1.015 (0.648–1.588)	0.949			2.875 (1.715–4.819)	** 0.000**	1.844 (1.032–3.295)	**0.039**
Obese vs overweight^c^	0.542 (0.320–0.918)	**0.023**	0.672 (0.345–1.308)	0.242	0.860 (0.685–1.080)	0.194			0.938 (0.626–1.406)	0.758		
Post-BS vs obese^d^	0.642 (0.317–1.301)	0.219			1.180 (0.756–1.842)	0.467			2.020 (1.421–2.870)	** 0.000**	2.191 (1.146–4.189)	**0.018**

^a^Adjusted for the significant risk factors associated with pregnant women weight status from the univariate analysis: nationality, maternal age, DM, parity and ETHN.

^b^Adjusted for the significant risk factors associated with pregnant women weight status from the univariate analysis: nationality, maternal age, parity and ETHN.

^c^Adjusted for the significant risk factors associated with pregnant women weight status from the univariate analysis: nationality, maternal age, DM, parity and ETHN.

^d^Adjusted for the significant risk factors associated with pregnant women weight status from the univariate analysis: nationality, DM, and parity.

Significant values are in **bold**. Abbreviations: cOR, crude Odds Ratio; aOR, adjusted Odds Ratio; CI, Confidence Interval; PET, Pre-eclampsia; PIH, Pregnancy-Induced Hypertension; PPH, Postpartum Hemorrhage; GDM, Gestational Diabetes; NICU, Neonatal Intensive Care Unit; ART, Assisted Reproductive Technology.

The results indicated that all groups, including post-BS, obese, and overweight women, had significantly higher likelihoods of cesarean delivery compared to women with normal weight, even after adjusting for the significant risk factors from the univariate analysis at stage one.

Post-BS women were significantly more likely to have preterm, low birthweight (< 2500 g), or stillbirth babies compared to normal weight women, and were more likely to have low birthweight babies (including term low birth weight) compared to overweight and women with obesity, even after adjusting for the significant risk factors from the univariate analysis at stage one.

Pregnant women with obesity were also more likely to have PET and induced labor compared to women with normal weight, and induced labor was a significant outcome for obesity versus overweight women as well even after adjusting for the significant risk factors from the univariate analysis at stage one (Table 4).

Overweight women were more likely to have assisted births and extremely premature babies compared to women with obesity, even after adjusting for the significant risk factors from the univariate analysis at stage one. Both women with obesity and overweight had significantly higher odds of GDM compared to normal weight and post-BS women, with women with obesity being more likely to have GDM than overweight women even after adjusting for the significant risk factors from the univariate analysis at stage one (Table 4).

## Discussion

In this study, we investigated the incidence of post-BS, and the factors associated with the post-BS group in comparison to other BMI weight status. In addition, we investigated the neonatal and maternal outcomes associated with obesity in parturient women, with a specific emphasis on those who conceived following BS. Unprecedented in Qatar, this research involved a classification of participants into four groups, leading to six unique comparisons. The study revealed that advanced maternal age, parity > 1, diabetes, and hypertension were significant risk factors, particularly in the context of post-BS.

Within three vital comparison groups (women with obesity vs. overweight, women with obesity vs. normal, and overweight vs. normal), our findings unequivocally demonstrated a heightened risk of maternal and neonatal adverse outcomes with increasing BMI index^37^. Noteworthy outcomes included gestational diabetes, caesarean deliveries, and labor induction, aligning seamlessly with extensive epidemiological studies and National Institute for Health and Care Excellence (NICE) guidelines for managing obesity in pregnancy^38–40^. Our findings contribute not only to the Qatari context but also provide a valuable benchmark for global discussions on the impact of obesity on pregnancy outcomes.

In the realm of post-BS comparison groups (BS vs. women with obesity, BS vs. overweight, BS vs. normal), a profound reduction in the risk of gestational diabetes among post-BS women stood out prominently. The significance of this reduction was stark when compared to both the women with obesity group with no history of BS and the overweight group with no history of BS. The findings also shed light on a significant reduction in assisted birth among post-BS women, an aspect that has been notably underexplored in existing literature.

Comparing our GDM reduction results with other meta-analyses revealed intriguing nuances. While our estimated reduction in the odds of GDM post-BS (aOR = 0.39, CI 0.29–0.54, P < 0.001) paralleled some studies, it also showcased variations compared to others^23,25,41^. This discrepancy emphasizes the importance of contextual factors and warrants further investigation into the underlying reasons for such variations. Notably, our study identified a paucity of epidemiological investigations on the five other comparison groups, signaling a critical gap in understanding the comprehensive spectrum of outcomes in post-BS women.

In addition, post-BS women showed a significant reduction in assisted birth compared to overweight (4% vs. 9%, aOR = 0.46, CI 0.21–0.99, P < 0.05). Numerous studies proved GDM risk reduction for women who conceived following BS^23,25,37,38,41–44^, however, a shortage of epidemiological studies concerning assisted birth specifically were identified^45^.

Despite the evident reduction in GDM risk, our study calls attention to the need for vigilant monitoring of post-BS women for potential complications inadequately mitigated by BS. Cesarean delivery and other risks, including term low birth weight, low birth weight, stillbirth, and preterm neonates, demand particular consideration. These results may be due to nutritional deficiency and rapid weight loss^44^.

Our findings concur with a substantial body of evidence indicating an increased risk of preterm deliveries, stillbirth, and low birth weight neonates among post-BS women^12,16,23,25,33,42,44,46–55^. However, the scarcity of studies on low birth weight among term babies underscores the imperative for further investigation. Conflicting results on cesarean delivery^42,56,57^ highlight the multifaceted nature of this outcome among post-BS women, necessitating extended research to unravel its underlying complexities. These findings indicate the need for extended research on Caesarean Section (CS) and BS complications that are not fully understood and may be related to other factors, such as changes in maternal anatomy, fetal growth patterns, or other severe maternal complications.

The study provides valuable insights, though it is important to acknowledge a few areas for future improvement. The retrospective design introduces biases and limits causal inferences, and the use of 2017 data may not fully capture evolving trends or current clinical practices and healthcare advancements. While the study includes multinational and multi-ethnic groups, generalising findings beyond the Qatari population requires caution. The study also lacks detailed data on the specific types of bariatric surgery, weight gain during pregnancy, and the time between BS surgery and pregnancy; factors that could influence outcomes. Additionally, the absence of information on post-BS conditions like hypoglycemia or dumping syndrome in the context of alterations in maternal glucose metabolism may overlook important aspects of maternal and neonatal health. Furthermore, differences in data availability suggest skewness, particularly with older mothers and those with conditions like hypertension, diabetes, and caesarean deliveries. This overrepresentation may impact the applicability of results to younger or lower-risk populations. Future research with updated datasets and more detailed variables will strengthen these findings and expand their applicability. To improve generalisability, future studies should also aim for more representative samples and employ prospective, longitudinal designs to provide a dynamic understanding of obesity in pregnancy. Exploring the sophisticated factors contributing to the variability in GDM reduction across different studies is vital. In addition, investigating under-researched areas, such as assisted birth and the relationship between nutritional deficits and newborn weight, will further enrich our understanding of bariatric surgery's impact on pregnancy.

The strength of our study lies in its pioneering nature as the first in Qatar to comprehensively examine various risk factors in pregnant women post-BS compared to women with obesity, overweight, and normal-weight counterparts. The detailed examination of multiple comparison groups enriches the existing literature on obesity and pregnancy outcomes.

## Conclusions

As obesity rates surge, our study highlights the inextricable link between obesity in women of childbearing age and adverse health conditions, particularly during pregnancy.

While BS proves instrumental in reducing obesity-related pregnancy complications, GDM, pregnancy-induced hypertension (PIH), and macrosomia, it introduces potential risks that may result in adverse outcomes for both mothers and babies. These risks include nutritional deficiencies, anemia, changes in maternal glucose metabolism, and the possibility of having children who are small for gestational age^58^. These must be addressed proactively, ideally during preconception counselling. The identified gaps in literature call for further research to elucidate the correlation between nutritional deficits and newborn weight, thereby enhancing the holistic understanding of pregnancy outcomes in this unique demographic.  Future studies should consider the interaction of genetic and environmental factors, utilising a precision medicine approach along with population health analyses^59–62^

## Data Availability

This is a research article and all data generated or analysed during this study are included in this published article. All inquiries should be directed to Nader Al-Dewik: naldewik@hamad.qa.

## Abbreviations

OI: Ovulation Induction
WWRC: Women's Wellness and Research Center
BMI: Body Mass Index
BS: Bariatric surgery
CS: Caesarean section
DM: Diabetes mellitus
GDM: Gestational diabetes mellitus
HTN: Hypertension
EHTN: Essential hypertension
LBW: Low birth weight
NICU: Neonatal Intensive Care Unit
PPH: Postpartum hemorrhage
PIH: Pregnancy-induced hypertension
ART: Assisted reproductive technology
LBWT: Low birth weight
PET: Pre-eclampsia
PP obesity: Postpartum obesity
